# Recent Advances in Magnetic Resonance Imaging for the Diagnosis of Liver Cancer: A Comprehensive Review

**DOI:** 10.3390/diagnostics15162016

**Published:** 2025-08-12

**Authors:** Faisal Alshomrani

**Affiliations:** Department of Diagnostic Radiology Technology, College of Applied Medical Science, Taibah University, Medinah 41477, Saudi Arabia; fshomrani@taibahu.edu.sa

**Keywords:** magnetic resonance imaging, hepatocellular carcinoma, liver cancer, imaging biomarkers, radiomics, diagnosis

## Abstract

MRI is a non-invasive imaging technique employed today in modern diagnostic medicine due to the fact it is capable of generating tissue architecture and function information with high image resolution without the use of ionizing radiation, unlike x-ray or CT scans. The advantages of MRI discussed in this review include better soft tissue contrast, the opportunity to perform imaging in different planes, and the ability to detect small changes in tissues, which helps to use MRI in many specialties, including cancer diagnosis and staging, as well as neurological and cardiovascular diseases. More particularly, this review aims to assess the contribution of MRI to the detection of liver cancer, especially HCC and ICC—the most frequent and aggressive types of pathology. Because of its high-resolution, MRI provides clear visualization of the small hepatic lesion and vascular mapping, which is crucial for early diagnosis and staging. It also reveals higher sensitivity and specificity than ultrasound and CT in identifying liver cancer dimensions and relations with system vasculature and a safer technique for patients who need many follow-up images. This is in addition to newer techniques that have been developed from MRI, which include the DWI, DCE-MRI, and MRE, all of which yield functional information concerning the perfusion of the tumor and the stiffness of the tissue, respectively, thus improving the diagnosis. Moreover, the application of artificial intelligence to MRI is improving lesion identification and cancer assessment, as well as patient outcome prediction, while relieving the burden of radiologists. Suggested improvements for future work include the combination of MRI with other diagnostic approaches, including circulating cell analysis and molecular imaging in managing liver cancer. Still, there is a limitation in MRI’s access globally, because scanners are expensive and unavailable in some parts of the world. Technological improvements and greater availability will extend MRI more as a valuable modality in the treatment of liver malignancies, more so for diagnosis and staging.

## 1. Introduction

Hepatocellular carcinoma (HCC), along with liver cancer, continues to be one of the most important health problems worldwide. Liver cancer ranked as the sixth most frequently found cancer worldwide, causing death by cancer to come in as the third most deadly cause in 2020. The survival rates benefit from early detection because this approach makes more patients available for curative procedures [1].

Currently, Magnetic Resonance Imaging (MRI) stands as the leading technology for liver cancer detection as a highly effective and non-invasive imaging technique that is extremely essential in today’s diagnostic medicine [2]. MRI delivers outstanding soft tissue contrast that allows precise evaluation of liver lesions to permit early diagnosis and accurate identification. The lack of ionizing radiation in MRI makes it a secure testing method that patients can use when needing repeated imaging evaluations. When MRI scanning becomes part of liver cancer screening programs, it raises the potential to find tumors at their earliest stage when patients achieve better results [3].

Radiologists get back to their initial state and release signals that the MRI scanner can pick to reconstruct them for higher-resolution images using computer algorithms [4]. Since fats and water are the major ingredients of the human body, MRI concentrates its scrutiny on the hydrogen atom because of its reasonably large magnetic moment in comparison to other nuclei [5]. The actual contrast in MRI comes from the differences in water and fat content of various tissues. Thus, it is possible to distinguish between muscles, fats, and connective tissues, as well as pathological conditions such as tumors [6]. MRI is technologically superior to other imaging methods, including CT scans and X-rays, in that it does not use ionizing radiation. Ionizing radiation can alter the DNA structure within cells and is cancerogenic, with the risk increasing with cumulative dose [7]. Hence, MRI is less hazardous for either extended or multiple applications and particularly applicable for sensitive groups such as children or pregnant women [8]. This also makes MRI a method of choice for serial evaluation and follow-up of chronic diseases or malignancies because of the cumulative doses of radiation involved [9].

MRI has some distinct features that set it apart from other more general imaging techniques essential for high-resolution imaging, especially in soft tissue contrast and cancer detection [10]. One major advantage is the ability to differentiate between the densities of different kinds of soft tissue, like muscles, ligaments, tendons, and tumors. This high contrast is due to variations in the relaxation characteristics of body tissues in terms of their water and fat fractions [11]. MRI also provides multi-planar imaging, which means it can provide images in axial, sagittal, coronal, and oblique locations without having to reposition the patient [12]. It is particularly important during the assessment of complicated areas of the body such as brain infarction, the spinal cord, and knee joints, as well as during pre-surgical planning that requires extreme accuracy in orientation [13]. MRI can also use several specialized sequences to encourage certain tissue characteristics or physiological processes that cannot be demonstrated with conventional imaging techniques. There are unique factors that make MRI different in the imaging diagnosis, as it remains a non-invasive technique—this means that the internal structure examination of the patient’s body does not require surgical treatment or biopsy, which in turn minimizes the risks, associated with the diagnosis, for patients [2]. MRI does not use ionizing radiation unlike in some imaging techniques, including CT scans and X-rays; therefore, it is beneficial when serial imaging is needed in cases of chronic diseases [14]. MRI is essential in a variety of diagnostic procedures due to its ability to produce the high level of detail and accuracy necessary when visualizing soft tissues and the functional capabilities of an organ [15]. When diagnosing neurological disorders, MRI serves as the primary imaging tool in brain and spinal diseases that include tumors, stroke, and multiple sclerosis, as well as degenerative disorders like Alzheimer’s and Parkinson’s diseases [16]. MRI scan images are higher in resolution than CT scan images; they are useful for early detection of those abnormalities and the implementation of surgical plans, notably in the head of the human body [15]. MRI is also important in musculoskeletal imaging, which is used to diagnose muscular, ligamentous, tendinous, and joint disorders, including ligament rupture, cartilage degeneration, and bone marrow disorders [17]. MRI is the preferred modality in diagnosing liver cancer due to its capacity to define the size of the liver lesion, establish vascularity, and distinguish between benign and malignant liver malignancy [18]. MRI’s high-contrast resolution, multisectoral imaging, and functional investigation features make its use mandatory in a lot of medical specialties, with an emphasis on oncologic imaging [19]. Given that MRS can offer specific, non-invasive studies of soft tissues without the use of radiation, the technique will continue to be an essential tool in diagnostic medicine [20]. As MRI technology constantly develops, it may be anticipated that it will contribute even more effectively to diagnostics, including cases of cancer.

## 2. Methodology

This review is compiled on the account of literature gathered using PubMed, Scopus and Web of Science and involved using such keywords as “liver cancer”, “MRI”, “radiomics, DCE-MRI”, “LI-RADS”, and “abbreviated MRI” to research articles published between the years 2019 and 2024. The studies which were published in peer journals in English and concerned study of diagnostic performance, promising MRI methods, AI use, and cost-effectiveness were considered.

Inclusion and Exclusion Criteria:

The purpose of this narrative review is to consider recent evidence regarding the diagnostic use of magnetic resonance imaging (MRI) for liver cancer, with a particular focus on new technologies and applications, including abbreviated MRI protocols, artificial intelligence (AI), radiomics, and standardized systems such as LI-RADS. The guiding research question is the following: What are the recent relevant developments of techniques based on MRI, and in particular AI and radiomics, with regard to improving diagnosis and clinical assessment of liver cancer? The review makes use of peer-reviewed original research articles and reviews primary research studies, as well as meta-analysis articles published between 2019 and 2024, as long as they focused on MRI, abbreviated MRI, AI, radiomics, or LI-RADS to deal with liver cancer. Only human-related studies in the English language were investigated. Studies covering only animals, those that relied purely on scanning other than MRI, non-peer-reviewed sources, and articles not relevant to liver-specific oncology were excluded. These objectives are to synthesize existing evidence, contrast the two methods of diagnostics, and outline innovations that enhance the level of accuracy, accessibility, and clinical usefulness of liver cancer imaging.

## 3. The Pathological Features of Cancer and Their Visualization by MRI

The detection and characterization of liver cancer heavily depends on magnetic resonance imaging (MRI) because this diagnostic tool provides advanced imaging capabilities, together with superior soft tissue contrast. Enhancements during recent times have upgraded both the diagnostic quality and functional evaluation capabilities of the imaging technology.

X-ray is not very useful in depicting soft tissues and is not very helpful in differentiation, while MRI is invaluable with respect to the depiction of pathological changes related to cancer [21]. MRI has the advantage of high contrast in soft tissues, meaning that tissues can be distinguished by their physiologic characteristics from normal structures and pathologic ones [22]. Tumors are biologically heterogeneous, and they have specific pathological and microstructural characteristics that distinguish them from normal tissues. These include more cell concentration that affects the tissue stiffness and the intensity of the signals in the images; alteration of the angioarchitecture that may result in tortuous, chaotic, and leaky blood vessels, resulting in uneven perfusion; and substantial extracellular matrix (ECM) remodeling. The ECM also changes biochemically and mechanically to take into account more deposition and crosslinking of collagen to modify tumor progression and to resist treatment. The tissue permeability and vascularity, as the hallmarks of influence, can be measured with the help of dynamic contrast-enhanced MRI (DCE-MRI), and based on this parameter, it will be possible to determine malignancy with the help of the functional features of the study [23].

**Figure 1:**
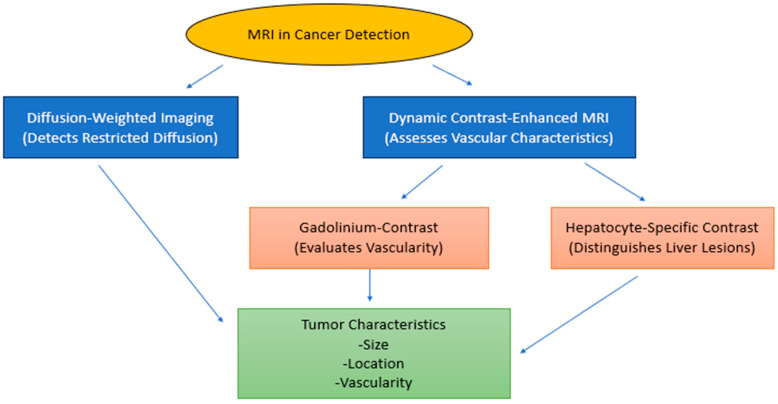
**Role of MRI in identifying cancer**. DWI is used to identify area of restricted diffusion, while DCE-MRI evaluates the vascular property. Other agents such as gadolinium and hepatocyte-specific contrast enhance the assessment of tumor vascularization while distinguishing liver lesions. The final output includes number, size, location, vascularity, and most important features that may be necessary to diagnose and treat cancer.

Because MRI provides high resolution in image acquisition due to multi-sequence imaging, MRI plays an important role in the diagnosis of oncological diseases based on tumor characterization [24], as illustrated in Figure 1.

Water molecule random Brownian motion detection through Diffusion-Weighted Imaging (DWI) reveals tissue cell count and cell membrane condition information. DWI detects water molecule Brownian motion to reveal information about tissue cellularity together with the condition of cell membranes during liver examinations. The heightened sensitivity of DWI enables medical staff to spot malignant lesions through its ability to identify restricted diffusion patterns which differ from normal liver tissue [25].

Dynamic contrast-enhanced MRI (DCE-MRI) measures tissue vascularity by applying contrast agents during the examination. The use of DCE-MRI techniques in liver cancer examinations permits physicians to analyze tumor blood flow patterns that help identify between benign and malignant tumor characteristics [26]. The latest developments in quantitative MRI include T1ρ and T2 mapping, which enable better examinations of liver tissue properties. The techniques deliver quantitative information about relaxation times to reveal tissue attributes and medical conditions [26]. Advanced MRI techniques, when used together, enable health professionals to provide better liver lesion evaluation, which enhances diagnostic precision and informs treatment strategies.

DWI estimates the water molecule displacement within tissues, and reduced water diffusion is often observed in malignant tumors because of their hypercellularity [27]. DCE-MRI, which documents the temporal enhancement profiles of tissues with contrast agents, provides valuable data concerning tumor perfusion and vascular properties [28]. HCCs are relatively hypervascular tumors, as the arterial phase of the scan shows early enhancement in the tumors, which washes out quickly in the venous phase, and this is the basis for detecting liver malignancies [29]. The technological inventions in imaging result in better cancer diagnosis, cancer staging, and assessment of cancer treatment response. Contrast agents are instrumental in making MRI as precise in detecting and characterizing tumors as it is. Contrast agents based on gadolinium are popular for MRI because they help visualize vessels and make differentiation between normal and pathological tissue possible [30]. These agents are particularly effective in cancer detection, since tumors exhibit highly unusual vascularization, as hypervascular tumors like HCC will have characteristic enhancement patterns when a gadolinium contrast agent is used [31].

This ability assists in the differentiation of malignant lesions from benign ones because the latter usually do not exhibit such sharp variations in vascular activity. In general, specifically in liver cancer imaging, there exist more specific types of contrast agents, such as gadoxetate disodium, more commonly referred to as Eovist or Primovist [30]. These agents are more preferable for uptake by hepatocytes, optimizing the evaluation of malignant and benign hepatic trauma [32]. Exemplary malignant tumors, with a focus on HCC, reveal decreased uptake of the hepatocyte-specific contrast and thus delineate as a hypointense (dark) area during the hepatobiliary phase of imaging, since malignant tumors lack functional hepatocytes [33]. However, non-neoplastic lesions such as focal nodular hyperplasia (FNH) usually expend or take up the contrast agent and are therefore easy to differentiate from malignant tumors [34]. This contrast specific to hepatocytes brings a great added value to MRI in the diagnosis of liver cancer, as it offers essential data for planning treatments and the overall prognosis. When used in conjunction with MRI’s sophisticated sequences, clinicians can obtain a detailed appreciation of tumor size, position, blood flow, and tissue type. Such detailed information is crucial for proper diagnostics, the establishment of stages of cancer, as well as for selection of the most appropriate therapeutic approach for the cancer patient.

## 4. Modern Approaches in Cancer Imaging Diagnosis

Diagnostic procedures used in cancer imaging today can be categorized in terms of the methodologies they employ, although each has a set of strengths and weaknesses. Of them, biopsy and histology retain their positions as the gold standards for cancer diagnosis [35]. This is an invasive process where tissue samples from suspicious lesions are taken for microscopy examination to allow pathologists to analyze cellular morphology to identify malignant cells [36]. In combination with imaging techniques, blood tests that determine the concentration of special proteins are gradually catching up with the stakes of malignancy. For example, alpha-fetoprotein (AFP) became a popular serum indicator of hepatocellular carcinoma (HCC) [37]. Raised AFP often can act as an early sign of liver cancer in individuals with liver disease [38].

**Figure 2:**
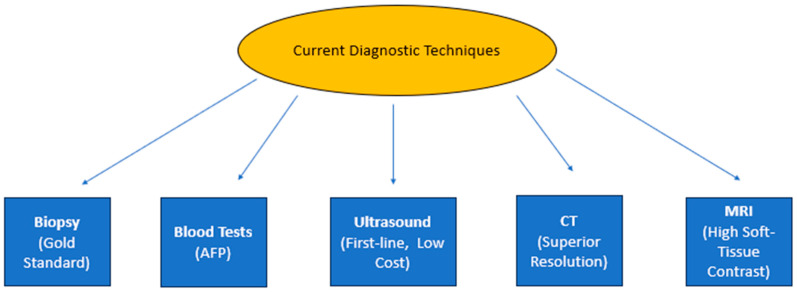
**Flowchart of the current diagnostic techniques in cancer imaging**. The central node specifies biopsy as the most accurate albeit invasive method, and biochemical tests, such as AFP for liver cancer, rank low on diagnostic efficacy but good on cost-effectiveness. At the same time, CT, with a high spatial resolution, and MRI, with soft tissue differentiation and multiple planes, are optimal for diagnosing cancerous tissues non-invasively.

Ultrasound is the most common imaging modality used to diagnose liver cancer and part of the standard staging evaluation because it is readily available, cost-effective, non-invasive, and does not use ionizing radiation [39]. Nonetheless, it lacks specificity in detecting early-phase tumors [40]. Still, CT scans present higher spatial resolution and are generally used for staging liver cancer. Computed tomography may show the size and the degree of invasion and visibility of metastasis to regional lymph nodes or other organs [29]. While having certain definite advantages, CT scans are based on ionizing radiation, raising questions concerning the further observation of cancer patients [41]. CT is less effective than MRI, where there is no proper contrast with soft tissue, and it also fails to differentiate between benign and malignant tumors [42]. With growing limitations of ultrasound and CT in routine oncology imaging, MRI is increasingly used to complement imaging in cancer diagnosis [43]. Through the provision of high soft tissue contrast, MRI is more accurate in tumor detection, particularly in small tumors, as well as the characterization of tissue, which is essential in the staging and management of cancer [44], as illustrated in Figure 2. MRI can better demonstrate the multi-planar anatomical imaging and functional status of the tumors themselves, including perfusion and diffusion characteristics—information for cancer detection [45]. Therefore, MRI has a special role in present-day oncology, especially in diagnosing and reviewing progress in liver cancer, where high-precision image formation affects therapy plans and results.

## 5. MRI in Liver Cancer Diagnosis

### 5.1. MRI in Diagnosis of Hepatocellular Carcinoma (HCC)

Hepatocellular carcinoma (HCC) is the most frequent and accounts for more than 80% of all primary liver cancers worldwide [46]. MRI is now widely used in the diagnosing and staging of HCC, and it offers better accuracy of small hepatic lesions than ultrasonography or CT [47]. The strengths of MRI in HCC diagnosis include its capacity to visualize the characteristic vascular signature of this cancer, including the hypervascularization during the arterial phase and the washout phenomenon during the venous phase [48]. Gadoxetate disodium (Gd-EOB-DTPA) serves as a prominent hepatocyte-specific contrast agent used in liver imaging, and it markets under Primovist in Europe and Eovist in the United States. Gadoxetate disodium functions as a solitary hepatospecific paramagnetic gadolinium-based contrast agent dedicated for MRI liver imaging. Medical professionals use this substance to interpret liver lesions [49]. The hepatobiliary gadolinium agent gadobenate dimeglumine releases 2–4% of its substance through biliary excretion, although its main route is renal elimination [50]. Researchers have conducted recent studies about the potential uses of dual-energy computed tomography (DECT) in liver examination. The multiparameter quantitative capability of DECT aids medical professionals in both early diagnosis and characterizing hepatocellular carcinoma (HCC) [51]. MRI is used in detecting intrahepatic cholangiocarcinoma (ICC).

ICC is a rare but highly malignant neoplasm that begins in the intrahepatic bile ducts, and MRI has an essential function in the identification and evaluation of ICC [52]. Unlike HCC, ICC does not demonstrate typical arterial phase hyper-perfusion and venous phase wash-in characteristics, although MRI affords excellent soft tissue contrast and the capability of detecting biliary lesions, peritumoral fibrosis, and other small tissue changes. MRI with MRCP is particularly beneficial in the delineation of the bile duct pathology non-invasively, helping in both diagnostic and local stages in ICC [53]. Table 1 provides a summary of the key MRI imaging techniques involved in the diagnosis of liver cancer, the various specific uses of each technique, as well as their most significant findings in diverse clinical applications.

Critical to this, ICC frequently coexists with biliary obstruction or ductal dilation. Furthermore, it is shown that DCE-MRI is beneficial to discern the enhanced pattern of ICC compared with other hepatic tumors, which is significant for proper treatment strategies [54]. MRI’s enhanced imaging therefore again offers significant information in the structural/functional anatomy of ICC and to a greater extent helps in early intercession and more specific treatment plans [55].

### 5.2. Comparison of MRI with Traditional Diagnostic Techniques

**Ultrasound (US)** is usually the first imaging modality employed in the evaluation and staging of patients with liver cancer because of its availability and affordability [56]. Ultrasound is less sensitive in soft tissue planes and resolution to characterize tumors, and early-stage lesions may not be seen [57]. However, MRI performs well in this aspect, which provides significantly better soft tissue contrast and the ability to visualize and characterize more minor lesions that might be beyond the sensitivity of the US, making it more accurate for the examination at the early stage [57].

**Computed Tomography (CT)** scanning is the other important method that is used in the diagnosis and staging of liver cancer [58]. First, for the detection of liver lesions, CT has good spatial resolution and a high sensitivity to detect tumors, but it uses ionizing radiation, which is not safe for patients for repeated scans and long-term follow-up cases like chronic liver diseases [59]. MRI does not employ radiation at all, and in addition to that, it provides better soft tissue contrast than CT, particularly in distinguishing between benign and malignant hepatic masses [60]. Table 2 summarizes the diagnostic accuracy measurements of regular MRI, abbreviated MRI, ultrasound, computed tomography (CT), and contrast-enhanced methods applied in liver cancer detection in terms of the chosen articles.

**Liver biopsy** continues to be the most definitive means of diagnosing liver cancer, especially when the results of imaging tests are unclear. Nevertheless, a biopsy is an invasive procedure that associates certain mortality risks, such as bleeding, infection, or tumor seeding [61]. It also has problems similar to any other diagnostic methods such as sampling errors and interobserver variability. MRI has high diagnostic sensitivity and specificity for the characterization of liver lesions [62].

**Figure 3:**
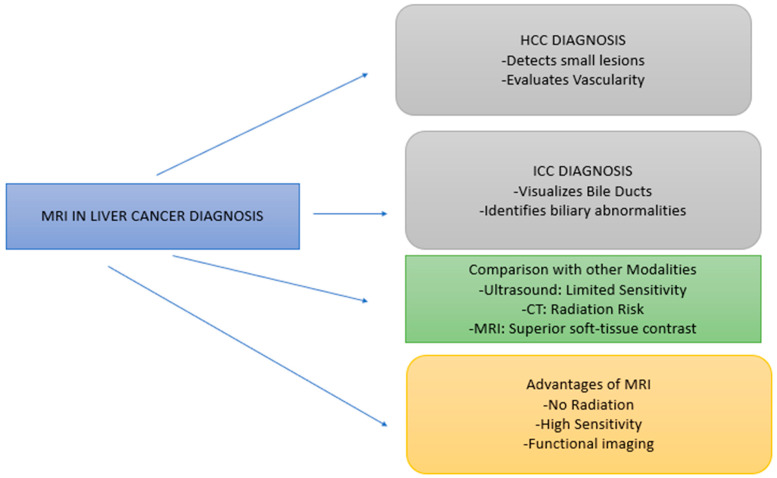
Demonstrates the sensitivity of MRI in the diagnosis of liver cancer, emphasizing its utility in the identification of HCC, and ICC over ultrasound and CT. The chart presents MRI as a method that helps visualize small lesions, evaluate vascularization, and offer functional imaging without using ionizing radiation.

MRI has some advantages over other diagnostic imaging technologies, which makes its use more appropriate for identifying and combating liver cancer, as illustrated in Figure 3. This technique has a stronger contrast with regard to soft tissues when compared to other types of images. MRI has terrific contrast resolution between various soft tissues and that makes it immensely valuable when such small liver tumors are present and helps to differentiate between benign and malignant ones. It also makes MRI an essential modality of choice in the early detection and management of liver malignant neoplasms. The other benefit of MRI is that it does not utilize ionizing radiation.

MRI is a technique that can also name functional imaging possibilities. Molecular imaging methods can be employed; for example, diffusion-weighted imaging (DWI), magnetic resonance spectroscopy (MRS), and magnetic resonance elastography (MRE) offer valuable information about tumor biology, tissue elasticity, and metabolism [63]. Most of these techniques are quite valid for the evaluation of fibrosis and cirrhosis, which are two important conditions that lead to liver cancer. DWI is focused on differences in cell density to help identify malignant tumors [64], while MRE enables non-invasive assessment of hepatic stiffness for diagnosing liver fibrosis [65]. Tumor DWI improves the contrast between malignant and benign lesions, as well as provides useful information about tumor analogous zone activity and treatment response early in the disease process by better defining water molecular motion within tissues [66]. One of them is magnetic resonance elastography (MRE) used for the evaluation of tissue elasticity, which is a key parameter in the differential diagnosis of liver fibrosis and cirrhosis—both of which are important risk factors for hepatocellular carcinoma (HCC) [67]. MRE provides a means to quantify liver stiffness without invasive intervention and provides more accurate information about the current state of the liver and its diseases compared to biopsies [68]. Normal and tumor-affected tissues differ, and reliable representative images of MRI play a major role in their explanation. T1- and T2-weighted images exhibit different changes in signal intensities, with the tumor areas being frequently hyperintense (T2) because of the presence of more water and hypointense (T1) because of necrosis and cysts [69]. Recent post-contrast DCE-MRI shows swift upstream and downstream contrast activity in cancerous tissues, as well as indicating altered vascular permeation and extensive perfusion compared to normal tissues, which experience less rapid and dispersed growth of activity. These radiological changes support the quantitative results and prove the biological basis of malignancy-related signal changes [70].

The application of AI in MRI analysis is revolutionary in diagnosing liver cancer. This affects special applications of AI, such as the ability to review sections of specialized imaging information for the detection of liver lesions, determining the characteristics of a tumor, or the consequences and evolution of the disease all in significantly less time than that required by clinical professionals [71]. Some of them can potentially decrease the role of human factors, improve the accuracy of diagnosis, and contribute to the planning of individual treatment. MRI is enhanced by artificial intelligence by the prospects of using a non-invasive technique for the early detection of liver cancers and the delivery of more personalized treatment options [72]. Prospective contributions of AI advances to MRI throughput, diagnostic reproducibility, and the provision of bespoke prognostic information in the treatment of liver cancer will be further enhanced as the underlying technology unfolds [73]. In combination, these innovations increase the accuracy and speed at which liver cancer is diagnosed, relieve the pressure on radiologists, and, potentially, will enhance the quality of treatment through earlier detection and intervention. Thus, MRI has now emerged as an essential imaging modality or a useful marker in the diagnosis and treatment of these liver cancers, including HCC and ICC.

### 5.3. Current Development and Future Trends in MRI for Liver Cancer

**Figure 4:**
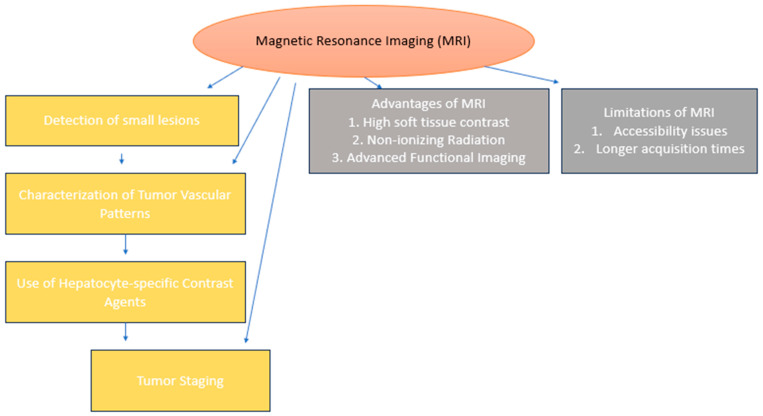
**A flowchart showing MRI’s positional role in diagnosing liver cancer**. The flowchart shows the progression from the detection of small lesions to the characterization of tumor vascular patterns and tumor staging. It also involves a brief description of the strengths and weakness of MRI, including high soft tissue contrast and the benefits of non-ionizing radiation—but at the same limited accessibility in comparison to other imaging technologies—and acquisition time.

Modern developments in MRI techniques have greatly improved the diagnosis, categorization, and treatment of liver cancer, as illustrated in Figure 4. Of these, DWI has turned out to be a very significant innovation that can help in the detection of cellular disparities within tumors. As DWI gives the number of water molecules by calculating the diffusion, it offers tissue characterization, tumor cell density, and microarchitecture, which are very important for the evaluation of the lesion aggressiveness of liver mass [74]. In addition, DCE-MRI is an essential tool in measuring tumor perfusion and vascularity as a means for distinguishing between benign and malignant tumors [75]. Another focal development is magnetic resonance elastography (MRE), a non-invasive technique used to evaluate the elasticity of the tissue. One of the conditions for which MRE is especially valuable is diagnosing liver fibrosis, which is the potential cause of hepatocellular carcinoma (HCC) [76]. Compared with other diagnostic methods, MRE is capable of determining the mechanical characteristics of liver tissue, which will enhance the identification of high-risk patients for cancer [65].

Dynamic contrast-enhanced magnetic resonance imaging (DCE-MRI) is a non-invasive imaging technology that measures blood perfusion of tissue and vascular permeability by imaging real-time variations in signal strength after the injection of gadolinium-labeled contrast agents. In contrast to the conventional MRI, which mostly serves as an anatomical imaging modality, DCE-MRI entails a functional imaging element due to the ability to measure parameters that include blood volume, vascular permeability, and extracellular volume. Such characteristics are particularly applicable in the diagnosis of inflammatory diseases, such as periodontal afflictions, in which the microvascular alterations identify the presence of the disease.

DCE-MRI has been used in periodontal applications to show images of gingival inflammation, differentiate active and chronic lesions, and measure response to treatment [77]. It can be used as a substitute for conventional diagnostic equipment since it can measure soft tissue perfusion. Nonetheless, its cost, limited availability, and poor resolution of the oral cavity limit the use of DCE-MRI to experimental or hospital-based studies.

Further improvement can extend its scope of personalized periodontal diagnostics, especially in combination with artificial intelligence and quantitative imaging biomarkers.

### 5.4. The Role of AI and Radiomics in Liver Cancer MRI

In addition to all these technical innovations, the use of artificial intelligence (AI) in MRI analysis is gradually finding its way into the industry [78]. Some applications of AI include the detection of liver lesions, evaluation of the progression of tumors, and tracking the patient’s prognosis by analyzing challenging image data [79]. These machine learning techniques can potentially enhance diagnostic capabilities in a way that would freely allow radiologists to come to decisions much more quickly, all while being more accurate. Therefore, extending the application of AI for real-time MRI can also contribute to the development of tailored treatment plans as well as point to particular imaging markers that are associated with patient reactions to treatment [80].

The use of artificial intelligence (AI) together with radiomics has transformed liver cancer MRI exams because it improves the capability to identify tumors and classify their type while assessing treatment outcomes. Modern machine learning (ML) and deep learning (DL) systems provide radiologists accurate and efficient detection of hepatocellular carcinoma (HCC), intrahepatic cholangiocarcinoma (ICC), and liver metastasis. AI-based systems demonstrate more than 90% accuracy in identifying benign from malignant liver lesions by processing multiparametric MRI data [81]. Through deep learning analysis of the MRI scans, the AI systems abstract automatic features, which enables them to detect complex imaging characteristics that exceed human perception abilities. A diagnostic refinement through automated systems becomes possible by processing T1-/T2-weighting, diffusion-weighted imaging (DWI), and contrast-enhanced MRI (CE-MRI) data points simultaneously. Research evidence shows that CNN technology surpassed human radiologists’ capacity to detect small HCC tumors, particularly during early disease stages [81]. Table 3 lists some clinical research on the application of artificial intelligence and radiomics-based models in interpreting MRI findings, whose accuracy in the classification of liver cancer has shown encouraging results. Biomarkers based on radiomics are quantitative, which are derived to consider medical images by employing sophisticated algorithms that describe the heterogeneity, texture, pattern, and shape of tumors. Some of these features are entropy, kurtosis, skewness, gray-level co-occurrence matrix (GLCM) values, and fractal dimensions, and they are non-invasively detected by MRI, CT, or PET scans. These biomarkers have been shown to be accurately predictive of tumor type, grade, therapy response, or patient outcome when studied based on machine learning models [82]. Radiomics facilitates the conversion of regular images to high-dimensional data, which assist precision medicine by opening up unknown patterns relating to underlying tumor biology.

The analysis method known as radiomics allows researchers to extract numerous quantitative imaging characteristics automatically from medical images for prognosis prediction. Radiomics-based biomarkers help physicians examine tumor heterogeneity and aggressiveness by studying the variations in tumor texture, shape, and intensity features. HCC recurrence risk, along with treatment response assessment, benefits from precise predictions achieved using radiomic signatures obtained from CE-MRI examinations [83].

With artificial intelligence (AI), the analysis of MRI images has been revolutionized, with the scans being analyzed fast, correctly, and with scan data being studied automatically. Advanced machine learning methods, specifically convolutional neural networks (CNNs), can explore intricate patterns within medical images that are unrecognizable to human eyes. As an example of this, researchers proposed a hybrid model with deep CNNs and a trained three-layer perceptron that helped diagnose cervical cancer with high accuracy [84]. In the same way, researchers also used the multi-threshold uniform local ternary pattern in classifying cell phenotypes in fluorescence microscopy images, which exemplifies the feature recognition ability of AI at the cellular level [85].

The conducted studies highlight the possibility of using AI to improve cancer diagnostics and achieve better sensitivity, specificity, as well as diminish the forces of interobserver variability. The continued study of AI-powered segmentation, classification, and prediction models is showing that these systems also have a future in clinical workflow.

Different clinical studies examine the implementation of AI algorithms in liver MRI to extract image data for therapy planning purposes and predict treatment choices. AI-based MRI algorithms achieve better diagnostic consistency based on findings from a current multi-center study when determining HCC detection and risk estimation across various imaging techniques [83]. AI systems integrated into liver MRI workflows give radiologists better capabilities while improving diagnosis quality and treatment solution development, as well as disease prediction abilities. AI research in liver cancer imaging requires better algorithms together with improved biomarkers, which need clinical verification to achieve its maximum usefulness.

Several applied AI advancements are expected to facilitate the diagnosis and treatment of liver cancer shortly with better prognosis and reduced burden for radiologists. Future studies in MRI of liver cancer will therefore involve the enhancement of multiparameter imaging MRI with other imaging methods like liquid biopsy and molecular imaging. Such an approach could greatly improve early diagnostic and surveillance capacities, which in turn enhance basic knowledge of tumor characteristics. In addition, newly generated contrast agents that work selectively with tumor characteristics or markers may enhance MRI in diagnosing liver cancer. Moreover, improvements in the cost-effectiveness of MRI will also be needed as a means of increasing the availability of this diagnostic tool to serve as the gold standard for liver cancer diagnosis in LMICs and other low-resource countries, as described above. Because of the way it can make costly techniques accessible for many patients at once, the prognosis of liver cancer patients could be enhanced, as well as patients’ survival rates and quality of life.

The abbreviated MRI (AMRI) has been identified as the possible alternative to full-sequence liver MRI, where the sequence is shortened, and the diagnostic efficacy remains the same or does not deteriorate. Other studies document similar sensitivity and specificity of detecting the hepatocellular carcinoma (HCC), especially with the use of the contrast enhanced sequences. AMRI is more efficient, low-cost, and possibly uplifts accessibility on regular surveillance programs [86]. The American College of Radiology designed the Liver Imaging Reporting and Data System (LI-RADS) to standardize the reading and documentation of liver imaging of patients at risk of HCC. It enhances the inter-reader agreement and supports multidisciplinary communication methods to classify the lesions using the possibility of malignant classification according to the MRI features [87]. The addition of LI-RADS to clinical processes improves the consistency of diagnosis.

The recent development in the field of AI, especially in deep learning and radiomics, has further complemented the diagnostic value of MRI, as lesions could be discovered, segmented, and classified autonomously. Convolutional neural network (CNN) AI models have demonstrated AUCs greater than 0.90 in differentiating malignant liver lesions and benign liver lesions [88]. Radiomic characteristics, including texture, shape, and intensity, can give us information on tumor heterogeneity and be used in risk stratification. Nevertheless, the main limitations pertain to generalizability, interpretability, and standardization of data. Although the diagnostic capabilities of MRI are high, cost, infrastructure, and expertise restrict the application of MRI, especially in low- and middle-income settings. Ultrasound, on the contrary, is still the primary choice because of its affordability and availability, although it is less sensitive. Abbreviated MRI or ultrasound-MRI hybridization would play an internationalization role according to comparative cost-effectiveness analyses [89].

## 6. Conclusions

The future of periodontal treatment is changing very fast because of the increasing need to have more effective, patient-friendly, and localized therapy. The conservative management of periodontal disease, including scaling and root planning, systemic antibiotics, antiseptic mouth rinses, etc., has been the mainstay of therapies but is usually associated with limitations, notably invasiveness, systemic toxicity, lack of efficacy in deep pockets, etc. On the contrary, recent delivery methods like nanoparticle-loaded topical gels are breakthroughs. The formulations offer extended and specific release of drugs, enhanced muco-adhesion, and tissue penetration through biofilms, in addition to boosting tissue regeneration and decreasing inflammation. They have, however, issues concerning retention of the gels in the oral cavity, standardization, safety-testing, and approval in medical usage.

In the diagnostics field, there are common uses of conventional probing and X-rays that can be considered rather primitive, since they do not have a high level of sensitivity in identifying any pathological changes at an early stage. New and more complex alternatives, such as DCE-MRI and optical coherence tomography, provide superior diagnostic image quality through the appearance of vascular or microstructural alteration, but they are very expensive and at present are not practical enough to be used routinely in a clinical setting. Whether it is the established tools and the emergent tools integrated and used in a balanced manner or the use of patient-specific adaptations to a cure, the future of periodontal care is probably going to be marked by these two. In the end, teamwork involving all material scientists, clinicians, and bioengineers is critical towards continuing to streamline these technologies and guarantee their entry to the clinic. Though the MRI approach is very sensitive and specific in the diagnosis of liver cancer, particularly where its ability is enhanced by contrast and complex imaging properties, the technology is more often limited by its expenses, accessibility, and inter-operator inconsistency. It is easy to access and efficient through shortened models of MRI and AI technology. However, surveillance usually makes use of ultrasound, since it is affordable and accessible. The application of the context of the patient, the place of patient care, and the sources where expertise may be accessed might be the most feasible route of diagnosis and provide a potentially multimodal route.

## Figures and Tables

**Table 1:** MRI findings in liver cancer diagnosis.

MRI Technique	Purpose	Findings
Diffusion-weighted imaging (DWI)	Assessing tissue cellularity.	Identifies highly cellular tumors and small dependent lesions
Dynamic contrast-enhanced magnetic resonance imaging (DCE-MRI)	Evaluating the amount, frequency, and ratio of blood circulation within the body.	It provides information on the nature of the tumor’s vascularization and enhancement characteristics.
Magnetic resonance elastography (MRE)	Measuring tissue stiffness.	Discriminates between benign and malignant growth due to the hardness of the tumor.
Hepatobiliary phase imaging (using agents like Gd-EOB-DTPA)	Looking for abnormalities associated with the liver and the ducts of the gland.	Enhances visibility of tumor masses in the liver as well as bile ducts.

**Table 2:** Comparative diagnostic accuracy of imaging modalities in liver cancer.

Study	Modality of Imaging	Sensitivity (%)	Specificity (%)	Key Notes
[31]	Contrast-enhanced MRI	90–94	89–93	Deep learning classifier improves HCC detection accuracy
[56]	Ultrasound (US)	60–75	70–85	Recommended for HCC surveillance in chronic liver disease
[38]	US + Alpha-fetoprotein	~65	~82	Cochrane meta-analysis supports combined approach for early diagnosis
[55]	Abbreviated MRI (AMRI)	83–88	87–91	Comparable to full MRI; useful for rapid HCC screening

**Table 3:** AI-based models for liver cancer detection using MRI.

Study	AI Model	Input Type	Performance	Key Notes
[3]	Deep CNN Classifier	Contrast-enhanced MRI	AUC~0.91	Multi-center study using CNN for lesion classification
[81]	Radiomics + AI	MRI Segmentation Maps	Variable	AI used for segmentation and detection in HCC
[83]	Prelim ML Models (Meta-analysis)	Clinical and Imaging Characteristics	AUC~0.89	AI model is a valuable instrument in the prediction of reoccurrence after HCC treatment
[79]	General AI Applications in HCC	Mixed (MRI + Clinical Data)	Not stated	Review of the diagnostic and therapeutic AI applications in the sphere of liver diseases

## Data Availability

No new data were created or analyzed in this study.

## Abbreviations

NMR	Nuclear Magnetic Resonance
DWI	Diffusion-Weighted Imaging
FNH	Focal Nodular Hyperplasia
AFP	Alpha-Fetoprotein
HCC	Hepatocellular Carcinoma
ICC	Intrahepatic Cholangiocarcinoma
DCE-MRI	Dynamic Contrast-Enhanced Magnetic Resonance Imaging
MRE	Magnetic Resonance Elastography
US	Ultrasound
CT	Computed Tomography
MRS	Magnetic Resonance Spectroscopy
AI	Artificial Intelligence
AMRI	Abbreviated Magnetic Resonance Imaging
LI-RADS	Liver Imaging Reporting and Data System
